# White matter and neurochemical mechanisms underlying age-related differences in motor processing speed

**DOI:** 10.1016/j.isci.2023.106794

**Published:** 2023-05-03

**Authors:** Amirhossein Rasooli, Hamed Zivari Adab, Peter Van Ruitenbeek, Akila Weerasekera, Sima Chalavi, Koen Cuypers, Oron Levin, Thijs Dhollander, Ronald Peeters, Stefan Sunaert, Dante Mantini, Stephan P. Swinnen

**Affiliations:** 1 Movement Control & Neuroplasticity Research Group, Group Biomedical Sciences, KU Leuven, Leuven, Belgium; 2 KU Leuven Brain Institute, KU Leuven, Leuven, Belgium; 3 Department of Neuropsychology and Psychopharmacology, Faculty of Psychology and Neuroscience, Maastricht University, 6200 MD Maastricht, the Netherlands; 4 REVAL Rehabilitation Research Center, Hasselt University, Diepenbeek, Belgium; 5 Murdoch Children’s Research Institute, Melbourne, VIC, Australia; 6 KU Leuven, Department of Imaging and Pathology, Group Biomedical Sciences, KU Leuven, Leuven, Belgium; 7 Department of Radiology, Athinoula A. Martinos Center for Biomedical Imaging, Massachusetts General Hospital, Harvard Medical School, Boston, MA, USA

**Keywords:** Physics magnetic resonance imaging, Neuroscience, Cognitive neuroscience

## Abstract

Aging is associated with changes in the central nervous system and leads to reduced life quality. Here, we investigated the age-related differences in the CNS underlying motor performance deficits using magnetic resonance spectroscopy and diffusion MRI. MRS measured N-acetyl aspartate (NAA), choline (Cho), and creatine (Cr) concentrations in the sensorimotor and occipital cortex, whereas dMRI quantified apparent fiber density (FD) in the same voxels to evaluate white matter microstructural organization. We found that aging was associated with increased reaction time and reduced FD and NAA concentration in the sensorimotor voxel. Both FD and NAA mediated the association between age and reaction time. The NAA concentration was found to mediate the association between age and FD in the sensorimotor voxel. We propose that the age-related decrease in NAA concentration may result in reduced axonal fiber density in the sensorimotor cortex which may ultimately account for the response slowness of older participants.

## Highlights

• Aging is associated with decreased level of NAA and FD in the SM1 cortex
• Aging is associated with decreased effector selection speed
• The association between age and reaction time is mediated by FD and NAA in SM1
• The association between age and FD in the SM1 is mediated by NAA

## Graphical abstract

**Figure:**
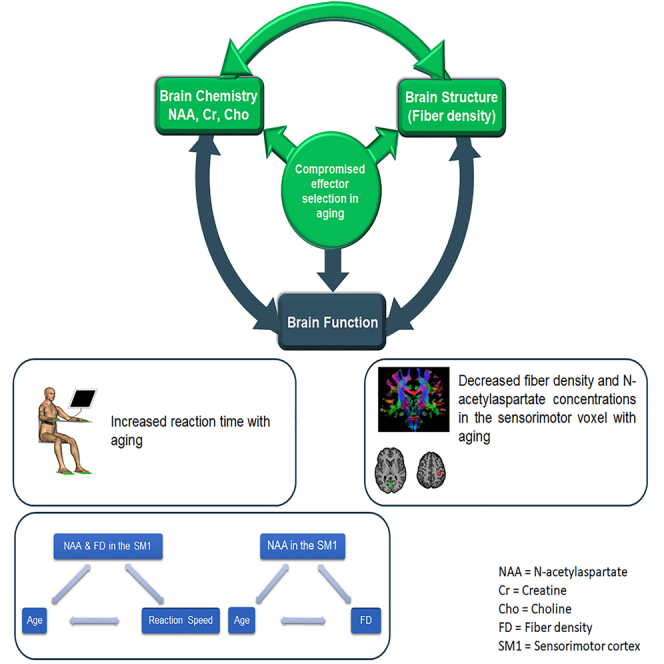

## Introduction

Aging is accompanied by changes in the central nervous system (CNS), which are in turn associated with cognitive, perceptual, and motor performance deficits. Understanding these age-related changes in the CNS is required for setting up interventions to delay or overcome these adverse effects, leading to the prolongation of a healthy and independent life in older adults.^1^ In view of the current demographic evolution of society, this is a critical research endeavor with high socio-economic relevance. Studies looking into the associations between age-related alterations in the brain and behavior may constitute a first step in tackling this research question.

Advances in neuroimaging techniques have played a pivotal role in understanding motor performance deficits in older adults. In this regard, age-related structural changes in the brain have been shown to be associated with bimanual/multi-limb motor performance deficits in older adults.^2^^,^^3^^,^^4^^,^^5^ Similarly, age-related changes in the neurometabolic content of the brain have been associated with performance decline in a wide spectrum of motor tasks.^6^^,^^7^ Alterations in structural and neurochemical measures can compromise behavioral performance either directly^8^ or indirectly through their interrelations.^9^ Revealing these interrelations is a daunting task that is central to current aging neuroscience. Here, we aim to reveal associations between white matter (WM) microstructure, neurochemicals, and motor behavior in younger and older adults.

A prominent technique for studying WM microstructural alterations in the brain is diffusion MRI (dMRI). Previous studies using a tensor-based model to derive diffusion metrics^10^^,^^11^^,^^12^ have suggested age-induced neurodegenerative changes in the brain.^13^^,^^14^^,^^15^^,^^16^ Even though these studies have provided valuable insights into age-related WM microstructural changes, they were limited by the assumption that the diffusion measured follows a Gaussian distribution.^17^ However, because WM voxels typically include multiple fiber populations with differing orientations, also known as “crossing fibers”,^18^ this assumption appeared too simplistic. To overcome this limitation, more sophisticated higher-order models have been developed.

One of these models is known as constrained spherical deconvolution (CSD), which can deal with crossing fibers by estimating the fiber orientation distribution (FOD) in each image voxel.^19^ The FOD represents the intra-axonal volume, i.e., the amount of WM axonal fibers within the voxel, along specific directions.^20^ This parameter, also known as apparent fiber density (FD), has been proposed as a proxy for the assessment of the microstructural organization of fibers within a voxel.^20^ Moreover, it has been widely used to study FD in a fiber population-specific approach.^21^^,^^22^ Reduced FD can indicate shrinkage or loss of axons in WM-related neurodegenerative diseases such as multiple sclerosis^23^while increased FD can designate an increased number of axons or diameter of the fibers in the process of neural development/plasticity.^24^ Hence, FD appears to be a relevant measure for the investigation of WM microstructural variations because of aging.^4^^,^^25^

Aging is often associated with changes in neurochemical concentrations,^7^^,^^26^^,^^27^^,^^28^^,^^29^^,^^30^which can be quantified using Magnetic Resonance Spectroscopy (^1^H-MRS). This technique allows *in-vivo* measurement of neurometabolite concentrations at various locations within the brain. The age-related alterations in neurochemical concentrations may either directly or indirectly play a role in the behavioral deficits (including motor function) by affecting various neural mechanisms, resulting in structural or functional changes in the brain.^7^^,^^27^^,^^31^^,^^32^^,^^33^ More specifically, N-acetyl aspartate (NAA), creatine (Cr), and choline (Cho) are of potential interest here because these compounds were suggested to play a potential role in WM-related processes as well as in motor performance, as discussed next.^7^^,^^33^^,^^34^

It has been speculated that ***N-acetyl aspartate*** (NAA) is a marker of neuronal viability, and hence, reduced NAA may imply neuronal loss.^29^^,^^35^ NAA is also involved in the myelination process through the donation of its aspartate group^36^^,^^37^^,^^38^ and it acts as an osmolyte in the osmoregulation procedure that is reflected in dMRI measures.^37^^,^^38^ Moreover, a number of studies have shown that NAA concentrations are associated with the intra-axonal compartment measure/metric of dMRI,^37^^,^^39^ indicating a possible link between NAA concentrations and WM-related processes. Regarding aging, even though a few studies reported that NAA concentrations remain stable across the human lifespan,^40^^,^^41^ recently emerging evidence suggests an age-related decrease in the concentrations of NAA in various brain regions, especially in the frontal areas.^26^^,^^42^

***Choline*** (Cho) has been suggested to act as a membrane metabolite^26^ and it also appears to be involved in WM-related neural processes, including gliosis and (de)myelination.^43^^,^^44^ Studies looking into the effects of aging on Cho have revealed heterogeneous results, with some reporting increased and others reporting stable concentrations of Cho across the lifespan.^26^ As fluctuations in the concentrations of Cho have been associated with membrane-related processes or variations in cell density,^26^ the age-related increase in Cho concentrations may imply membrane breakdown or turnover.^44^^,^^45^

***Creatine*** (Cr) is involved in neuronal energy homeostasis. It also acts as an osmolyte involved in the process of preserving cell volume and fluid balance.^44^^,^^46^ This volume balance is reflected in the intra-axonal compartment measure/metric of the dMRI signal, indicating a potential association between FD and Cr concentrations. Cr is suggested to be stable across the lifespan and it is often considered an internal reference for the whole spectrum of metabolites. However, recent investigations reported changes in the Cr concentrations in association with age.^7^^,^^47^^,^^48^ The age-related decline in Cr concentrations may lead to compromised energy homeostasis in neurons.

Overall, the limited number of studies so far appear to suggest that NAA, Cr, and Cho are associated with WM-related neural processes, e.g. myelination, gliosis, and osmoregulation, especially in the normal aging process. This motivated us to investigate the potential associations between WM microstructural organization, assessed via FD, and the above-mentioned neurometabolites across the lifespan. With respect to the motor performance domain, it has been shown that lower NAA concentrations in the sensorimotor cortex are associated with poorer performance in visuomotor functions, as tested by means of instrumented bimanual coordination as well as the Purdue pegboard tasks,^7^ with NAA being considered as an indirect marker for underlying GM/WM deteriorations.^7^ However, the association between NAA and WM microstructural properties remains to be investigated. In addition, it is still unclear for what range or type of motor tasks these associations between neurochemical concentration and behavioral performance are most noticeable.

**Figure 1:**
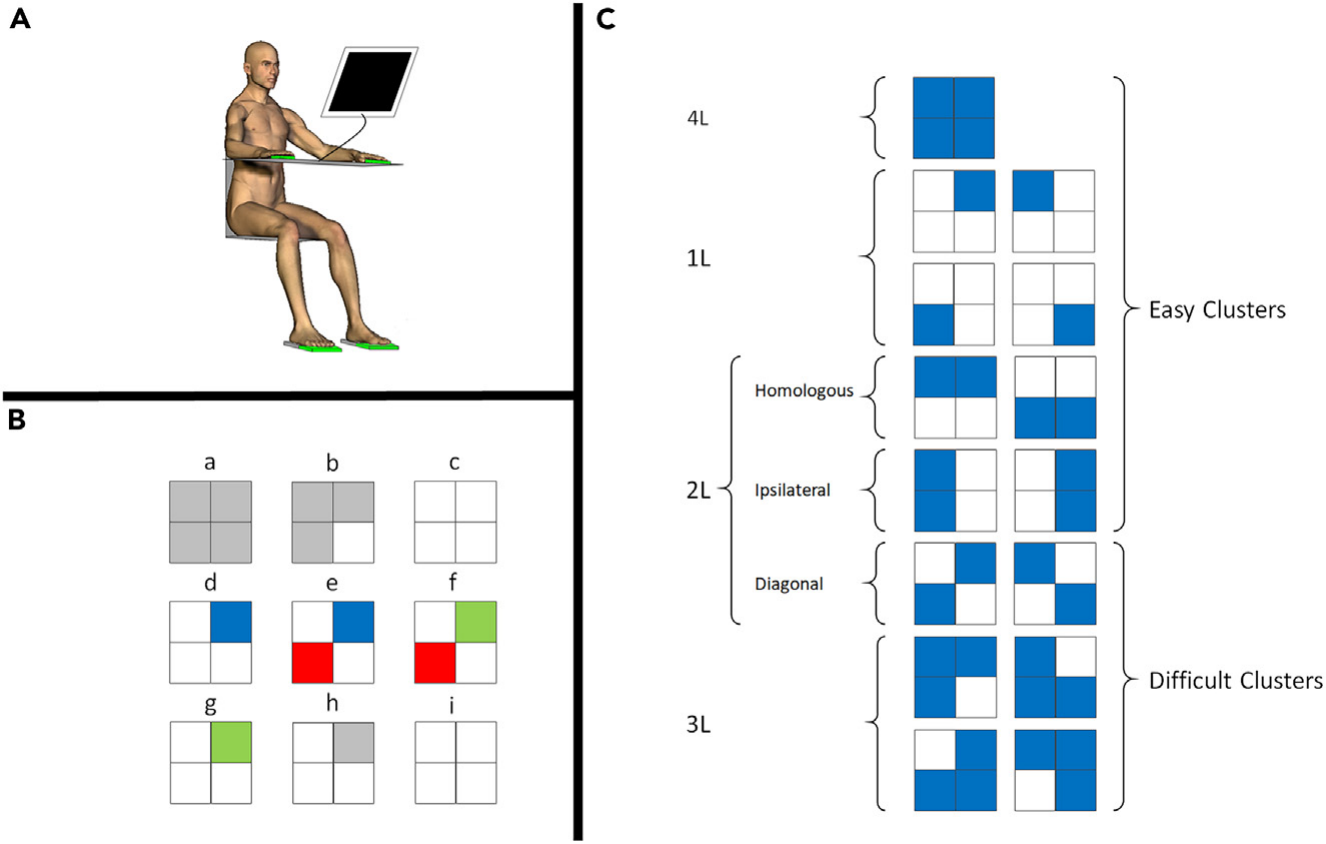
Behavioral task description (A) Experimental setup. Participants are seated in front of a screen, with their forearms resting on a table and their fingers and forefeet on tablets. (B) Exemplar MLRT task trial procedure. Top squares represent stimulus cues for left and right hands. Bottom squares represent cues for left and right feet, respectively. (B-a), When the limb segments are not connected to the tablets, the corresponding squares are gray. (B-b), They turn white as soon as the corresponding limbs contact the tablets. (B-c), The setup is ready for a trial when all of the limbs are in contact with their corresponding tablets. (B-d), After a randomly varying time ranging from 500 to 1000 ms, the stimuli are presented by blue squares, indicating the limbs that should be released as quickly and correctly as possible. (B-e), If the participant releases the incorrect limb, the corresponding square(s) turn(s) red. (B-f), If the participant lifts the correct limb(s), the corresponding square(s) turn(s) green. (B-g), A trial is not validated until the response is fully correct, that is, without any red square on the screen. (B-h), As soon as the trial is validated, the green squares turn back to gray. (B-i), Participants have to reposition all limb segments on the tablets to start a new trial. (C) Different stimulus modes and clusters: the 15 possible modes are grouped into 6 clusters (1L, 2L-Homo, 2L-Ipsi, 2L-Diag, 3L, and 4L) based on the number of limbs to be recruited (1, 2, 3, or 4) and the coupling/decoupling interactions involved. Previous studies^5^^,^^49^ have shown that 2-L Diag and 3L stimulus clusters are relatively more difficult to perform than the other stimulus clusters. Adapted with permission from.^49^

**Figure 2:**
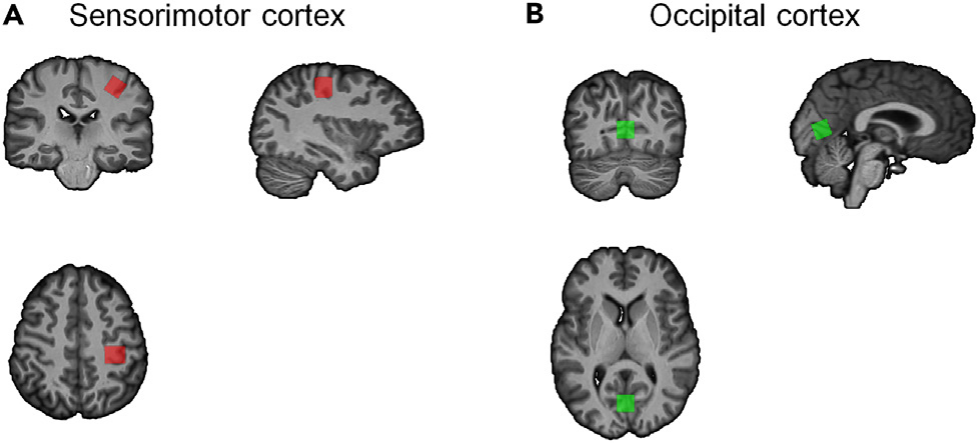
MRS voxels for an exemplary participant are overlaid on the T1 image (A) left sensorimotor voxel, (B) occipital voxel. Images are in the radiological view.

**Figure 3:**
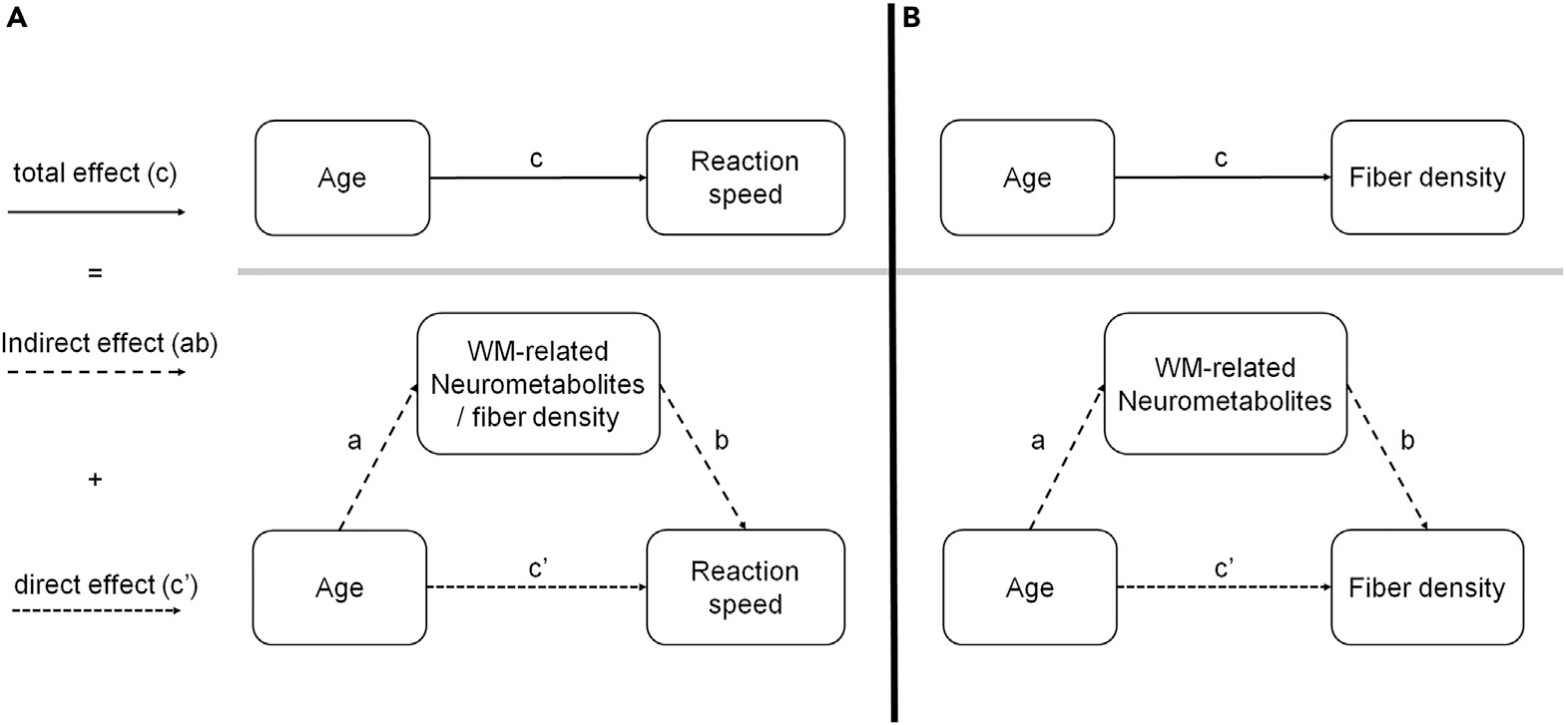
Three separate investigated mediation models (A) Two separate mediations are used to determine whether the age-related differences in reaction speed are mediated by: (1) Changes in the concentrations of WM-related neurometabolites or (2) the WM fiber density. (B) One mediation model is used to investigate whether the age-related differences in FD are significantly mediated through the decline of WM-related neurometabolites. In both A and B panels, the total effect of the independent variable on the dependent variable, the effect of the independent variable on the mediator, the effect of the mediator on the dependent variable controlling for the independent variable, and the residual effect of the independent variable on the dependent variables controlling for the mediator are indicated as c, a, b, and c’, respectively.

In this study, we aimed to investigate the effect of aging during the performance of a simple multi-limb reaction time (ML-RT) task (Figure 1) as a proxy for reaction speed^49^ with different levels of difficulty. Furthermore, we targeted the microstructural and neurochemical properties of the left sensorimotor cortex as a prominent region directly involved in the execution of this task, while the occipital cortex served as a comparison region because of its less critical role in motor execution (Figure 2). Moreover, prior to each trial, visual cues were provided about the upcoming stimuli. This pre-cuing allowed the participants to plan their actions in advance, thereby reducing the visual processing demands during RT performance. We hypothesized that aging is associated with a significant decline in both structural and neurochemical properties of the sensorimotor cortex on the one hand and behavioral (ML-RT) performance on the other hand. In addition, we hypothesized that the aforementioned measures of WM microstructure and neurochemicals mediate the aging effect on ML-RT performance (Figure 3). Finally, we hypothesized that the degenerative effects of aging on microstructural white matter properties of the brain might be mediated by the proposed neurometabolites.

## Results

### Age-related changes in reaction speed

#### Reaction time (RT)

**Figure 4:**
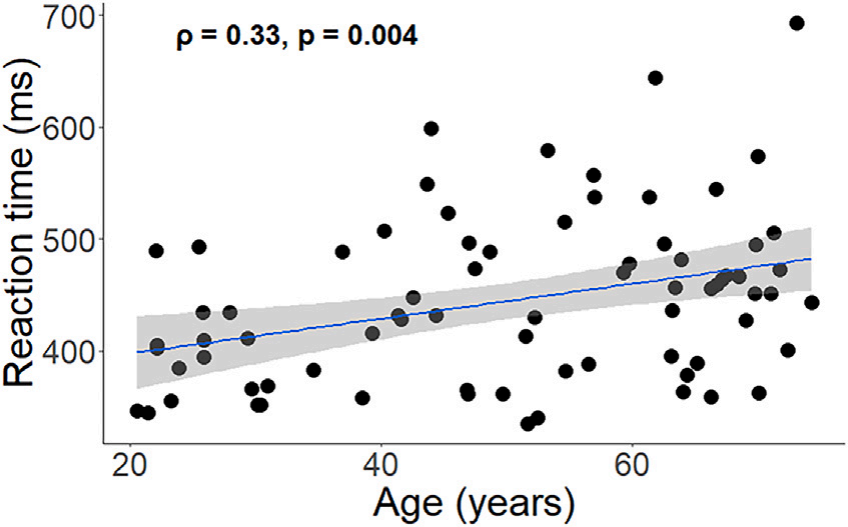
Association between age and motor reaction time (n = 76) A linear regression line with the 95% CI is added for illustration purposes. ρ = Spearman’s rho; ms = millisecond.

The correlation between age and RT was significant (ρ = 0.33, p = 0.004) indicating that increasing age is associated with increasing RT (Figure 4). Thus, older participants were generally slower in their multi-limb reaction performance than young adults.

### Age-related changes in neurochemical and microstructural measures

#### Fiber density (FD)

**Figure 5:**
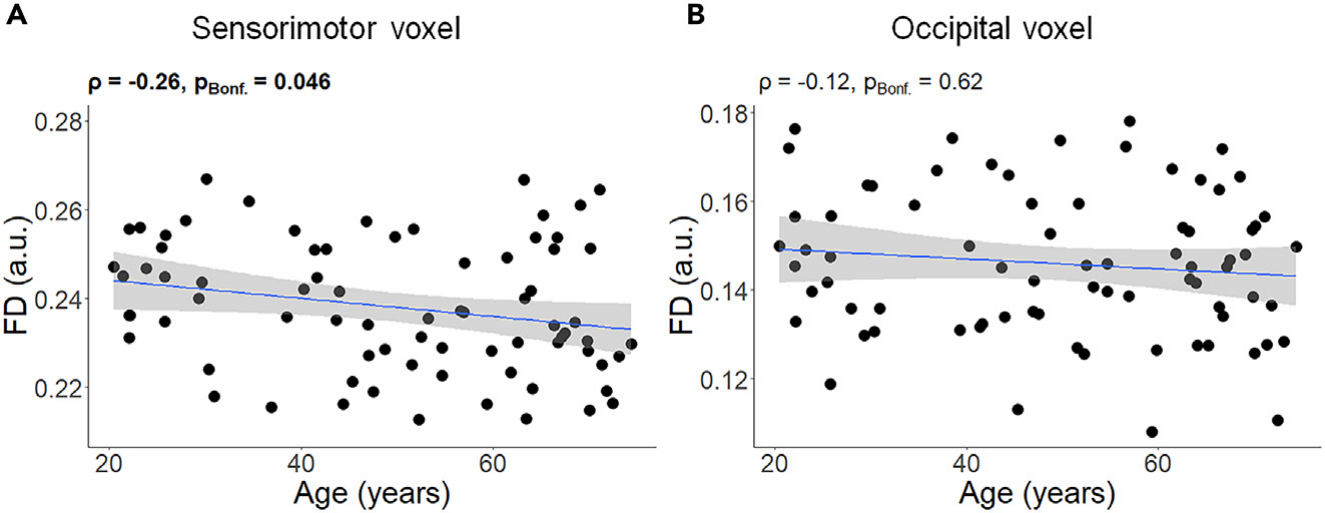
Association between age and FD (n = 76) (A) results in sensorimotor, and (B) results in occipital voxel. A linear regression line with the 95% CI is added for illustration purposes. a.u. = arbitrary unit; ρ = Spearman’s rho; pBonf. = p after Bonferroni’s correction for 2 comparisons (Significant correlation is indicated in bold); FD = Fiber density.

A negative correlation was found between age and FD in the SM1 voxel (ρ = −0.26, p_Bonf._ = 0.046) implying that the fiber density within the sensorimotor voxel decreased with increasing age (Figure 5A). In contrast, the association between age and FD in the Occ voxel was not significant (ρ = −0.12, p_Bonf._ = 0.62; Figure 5B).

#### WM-related neurometabolites

**Figure 6:**
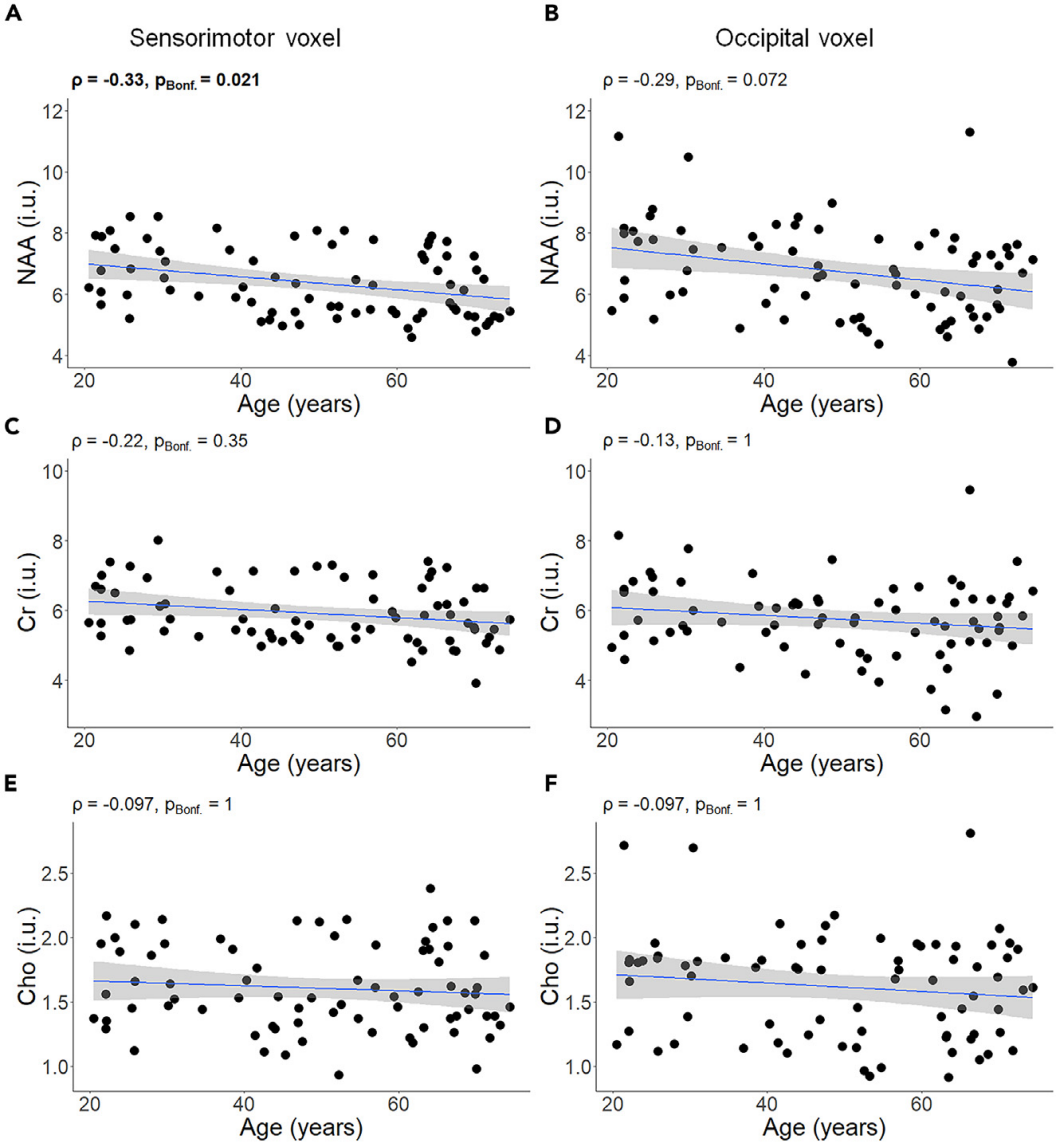
Association between age and concentrations of the WM-related neurometabolites NAA, Cr, and Cho (n = 76) (A, C, E) results in sensorimotor voxel, and (B, D, F) results in occipital voxel. A linear regression line with the 95% CI is added for illustration purposes. i.u = institutional unit; ρ = Spearman’s rho; pBonf. = p after Bonferroni’s correction for 6 comparisons (Significant correlation is indicated in bold); NAA = N-acetyl aspartate; Cr = Creatine; Cho = Choline.

Average spectra obtained from the SM1 and Occ voxels are presented in Figure S1. A significant negative association was found between age and NAA concentrations within the SM1 voxel (ρ = −0.33, p_Bonf._ = 0.021; Figure 6A) while this relationship was not present for either Cr (ρ = −0.22, p_Bonf._ = 0.35; Figure 6C) or Cho (ρ = −0.097, p_Bonf._ = 1; Figure 6E). This indicates that aging is accompanied by a significant decline in NAA concentrations in the SM1 area, whereas Cr and Cho concentrations remained stable. Similar relations were investigated in the Occ voxel. Although a negative correlation between age and NAA concentrations was present at uncorrected level (ρ = −0.29, p = 0.012; Figure 6B), this association did not survive Bonferroni’s correction for multiple comparisons (p_Bonf._ = 0.072). Moreover, similar to the SM1 voxel, associations between age and Cr (ρ = −0.13, p_Bonf._ = 1; Figure 6D) and Cho (ρ = −0.097, p_Bonf._ = 1; Figure 6F) concentrations were not significant in the Occ voxel, indicating the lack of evidence for age-related differences in concentrations of these metabolites in the Occ cortex in this sample.

### Association between brain metrics and reaction speed

#### Fiber density (FD)

**Figure 7:**
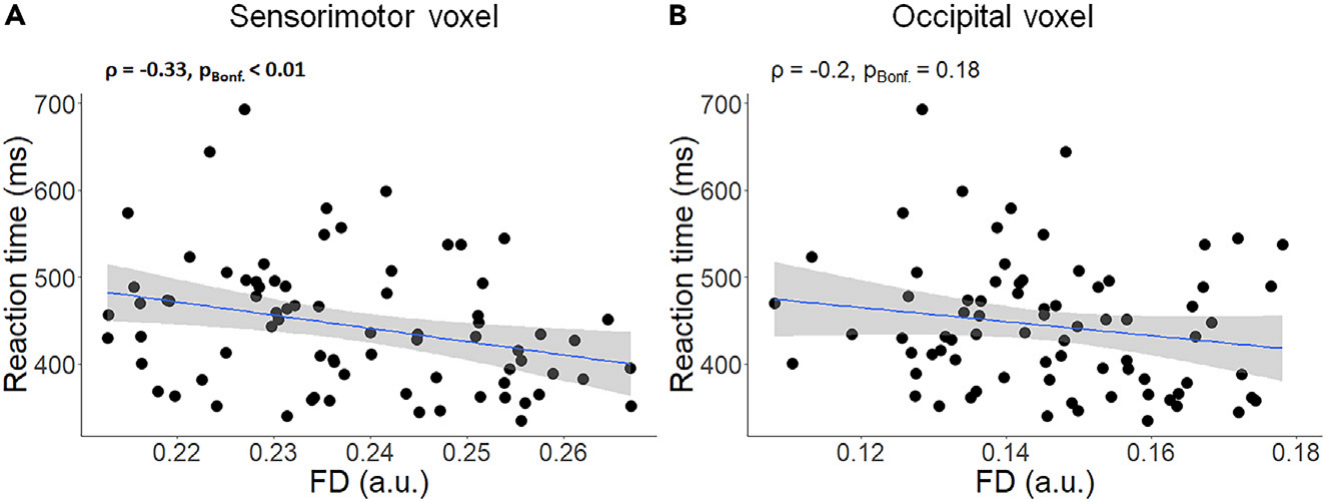
Association between reaction speed and fiber density (n = 76) (A) results in sensorimotor voxel, and (B) results in occipital voxel. A linear regression line with the 95% CI is added for illustration purposes. ρ = Spearman’s rho; pBonf. = p after Bonferroni’s correction for 2 comparisons (Significant correlation is indicated in bold); FD = fiber density; ms = millisecond; a.u. = arbitrary unit.

In the SM1 voxel, FD was negatively correlated (ρ = −0.33, p_Bonf._< 0.01) with RT (Figure 7A). Hence, participants with lower FD in the SM1 area were slower in reaction speed (RT). In contrast, the association between RT and FD was not significant in the Occ voxel (ρ = −0.2, p_Bonf._ = 0.18, Figure 7B).

#### *WM-related* neurometabolites

**Figure 8:**
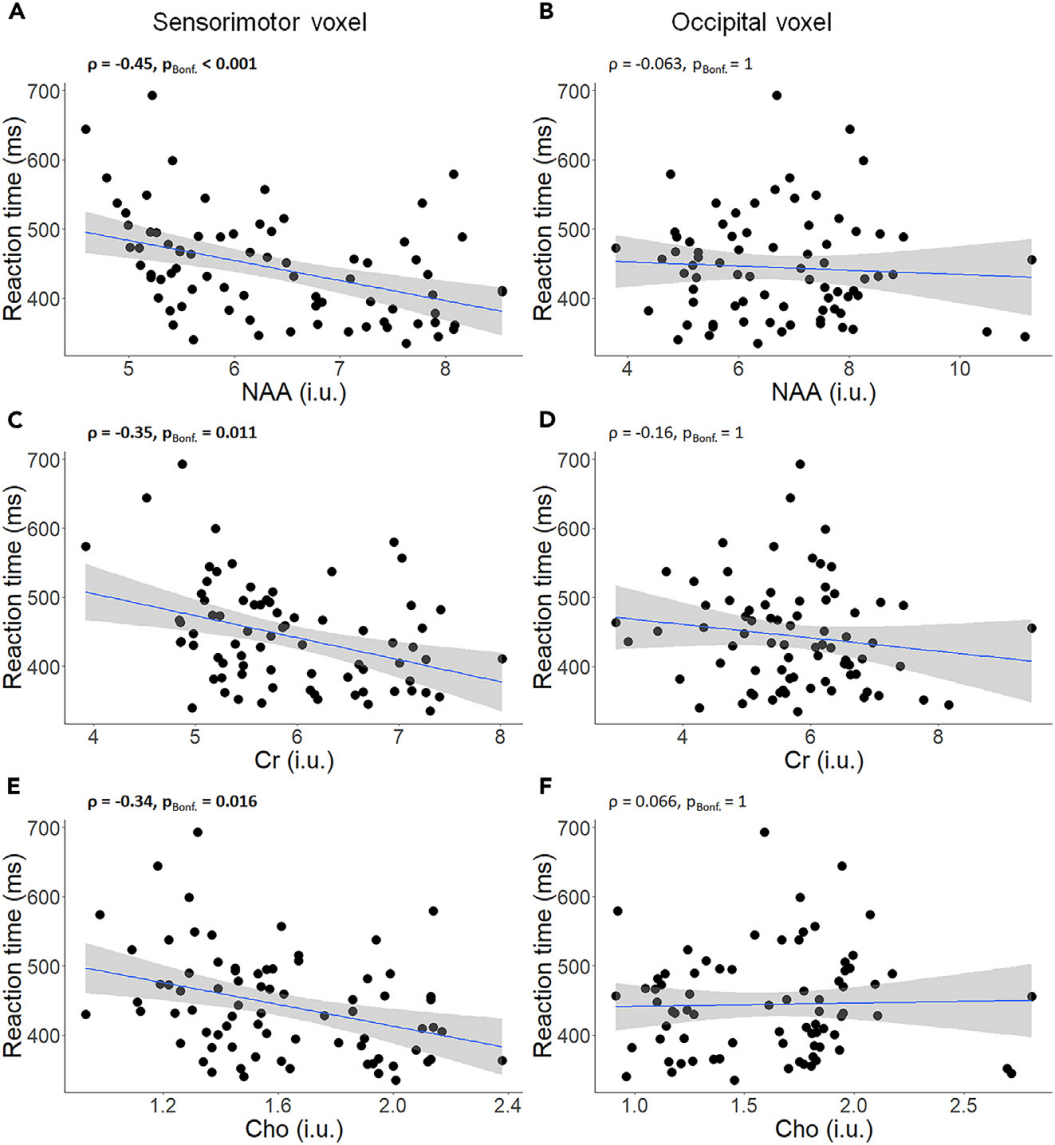
Associations between reaction speed and concentrations of WM-related neurometabolites (NAA, Cr, and Cho) (n = 76) (A, C, E) results in sensorimotor voxel, and (B, D, F) results in occipital voxel. A linear regression line with the 95% CI is added to each graph for illustration purposes. ρ = Spearman’s rho; pBonf. = p after Bonferroni’s correction for 6 comparisons (Significant correlations are indicated in bold); ms = millisecond; i.u. = institutional unit; NAA = N-acetyl aspartate; Cr = Creatine; Cho = Choline.

In the SM1 voxel, the concentrations of NAA, Cr, and Cho were negatively associated with RT (NAA: ρ = −0.45, p_Bonf._< 0.001; Cr: ρ = −0.35, p_Bonf._ = 0.011; Cho: ρ = −0.34, p_Bonf._ = 0.016; Figure 8A). Hence, lower concentrations of these metabolites were associated with higher RT, indicating slower motor reaction. Conversely, no significant associations between RT and neurometabolite concentrations were observed in the Occ voxel (NAA: ρ = −0.063, p_Bonf._ = 1; Creatine: ρ = −0.16, p_Bonf._ = 1; Choline: ρ = 0.066, p_Bonf._ = 1; Figure 8B). To ensure that the associations between the neurometabolite concentrations and reaction speed are not affected by age-related atrophy, we further investigated the relationship between the CSF fraction within each voxel (as a surrogate measure of age-related atrophy) and reaction time. These analyses revealed no significant correlations between reaction time and the CSF fraction in the Occ voxel (ρ = −0.017, p_Bonf._ = 1) and SM1 voxel (ρ = 0.23, p_Bonf._ = 0.084).

### Mediation analysis

Considering that the aging effect was observed for both FD and NAA concentrations in the SM1 voxel, and not in the Occ voxel, we proceeded with the mediation analysis of only the former voxel.

#### Age-reaction speed relation mediated through FD

**Figure 9:**
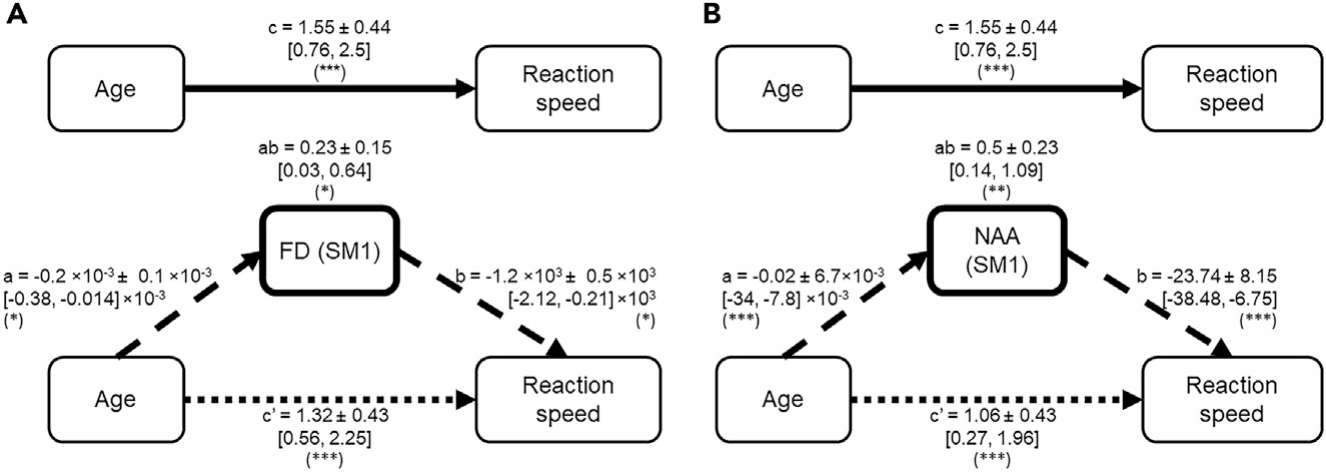
Results of standard three-path mediation analysis between age, reaction speed, and FD/NAA (A) Fiber density (ab = 0.23 ± 0.15, 95% CI = [0.03, 0.64]) and (B) NAA concentrations (ab = 0.5 ± 0.23, 95% CI = [0.14, 1.09]) significantly mediated the age-related increase in RT. The mean ± SE and [95% CI] path coefficient is shown. (∗) = p < 0.05, (∗∗) = p < 0.01, (∗∗∗) = p < 0.001. FD = fiber density; NAA = N-acetyl aspartate; SM1 = sensorimotor voxel.

Constructing a mediation model using age, FD, and RT, as independent, mediator, and dependent variables, respectively (Figure 9A), it became evident that the age-related decline in reaction speed (mean ± SE; c = 1.55 ± 0.43, 95% CI = [0.79, 2.5]) is mediated through FD (ab = 0.23 ± 0.15, 95% CI = [0.03, 0.64]). Hence, the indirect path of this mediation indicates that aging resulted in a reduction in FD (a = −0.2 ×10^−3^ ± 0.1 ×10^−3^, 95% CI = [-0.38, −0.014] ×10^−3^) which in turn led to an increase in RT (b = −1.2 ×10^3^ ± 0.5 ×10^3^, 95% CI = [-2.12, −0.21] ×10^3^). Notably, the significant direct path c’ (1.32 ± 0.43, 95% CI = [0.56, 2.25]), indicates that the observed mediation was rather “partial” than “complete”.

#### Age-reaction speed relation mediated through NAA

Similar to FD, results of the mediation analysis (Figure 9B) showed that age-related decline in reaction speed (c = 1.55 ± 0.43, 95% CI = [0.79, 2.5]) is mediated through NAA (ab = 0.5 ± 0.23, 95% CI = [0.14, 1.09]). Hence, the indirect path of this mediation indicates that aging resulted in a reduction in NAA concentrations (a = −0.02 ± 6.7×10^−3^, 95% CI = [-34, −7.8] ×10^−3^) which was associated with an increase in RT (b = −23.74 ± 8.15, 95% CI = [-38.48, −6.75]). Moreover, the significant direct path c’ (1.06 ± 0.43, 95% CI = [0.27, 1.96]), shows that this mediation is also “partial”.

#### Age-FD relation mediated through NAA

**Figure 10:**
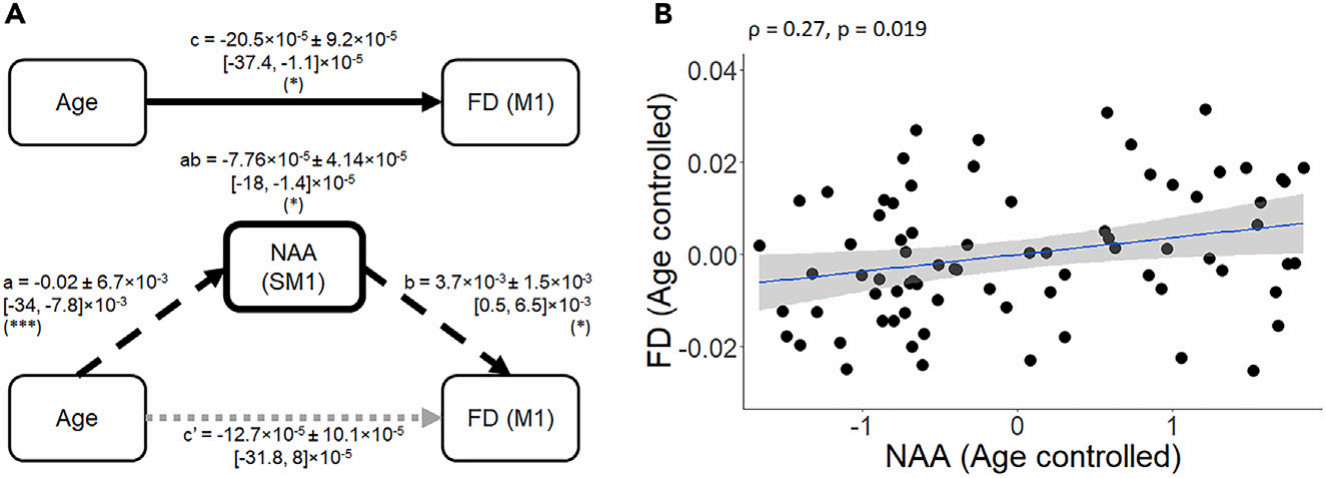
Results of standard three-path mediation analysis berween age, NAA, and FD (A) Concentrations of SM1 NAA significantly mediated the age-related differences in FD (ab = −7.76 × 10-5 ± 4.14×10-5, 95%CI = [-18, −1.4] ×10-5). The mean ± SE and [95% CI] path coefficient is shown. (∗) = p < 0.05, (∗∗∗) = p < 0.001. SM1 = Sensorimotor voxel. (B) Partial Age-controlled Spearman’s correlation between FD and NAA concentrations in the sensorimotor cortex (path b). A linear regression line with the 95% CI is added to each graph for illustration purposes. ρ = Spearman’s rho; FD = Fiber density; NAA = N-acetyl aspartate.

Results of the mediation analysis (Figure 10) showed that the association between age and FD (c = −20.5 × 10^−5^ ± 9.2×10^−5^, 95% CI = [-37.4, −1.1] × 10^−5^) was mediated through the indirect path via the NAA (ab = −7.76 × 10^−5^ ± 4.14×10^−5^, 95% CI = [-18, −1.4] × 10^−5^). Hence, the indirect path of this mediation indicates that aging resulted in a reduction in the NAA concentrations (a = −0.02 ± 6.7×10^−3^, 95% CI = [-34, −7.8] × 10^−3^) which was associated with a decrease in FD (b = 3.7 × 10^−3^ ± 1.5×10^−3^, 95% CI = [0.5, 6.5] × 10^−3^; see also Figure 10B). Moreover, the non-significant indirect effect (c’ = −12.7 × 10^−5^ ± 10.1×10^−5^, 95% CI = [-31.8, 8] × 10^−5^) indicates a “complete” rather than a “partial” mediation.

## Discussion

We investigated age-related differences in reaction speed and its underlying neural mechanisms using a multi-modal neuroimaging approach. As such, using dMRI and MRS, we respectively found a decrease in apparent fiber density (FD) and N-acetyl aspartate (NAA) concentrations with advancing age. Of interest, these results were only present in the sensorimotor and not in the comparison (i.e., occipital) voxel. Regarding the reaction speed, we found a significant increase in RT with aging. Moreover, this association was partially mediated by both fiber density and NAA concentrations in the sensorimotor voxel. Finally, we found that the age-related decline in fiber density was significantly mediated by the NAA concentrations in the sensorimotor cortex.

### Effect of age on reaction speed

A large body of research has revealed a decline in motor performance as a function of aging. Along the same line, using bimanual tracking and Purdue pegboard tasks, a performance decline in older adults compared to their younger counterparts has been shown.^7^^,^^50^ However, the latter tasks are more focused on motor coordination between the two hands. Here, we utilized a multi-limb reaction time (ML-RT) task to investigate the coordination between different limbs and to focus on reaction speed. Reaction speed is normally quantified by measuring reaction time. Previous research on the ML-RT task led to the proposition of a model of coupling and decoupling between the limbs.^49^ This model quantifies the motor reaction according to two process levels, i.e., (1) the “selection” process level which refers to central processing to select the proper combination of effectors among the possible options, and (2) the “recruitment” level which executes the response with the selected effectors. In previous work, this task was utilized to investigate aging deficits in the “selection” process of multi-limb reaction. It was evidenced that older adults spent more time on effector selection compared to younger adults.^3^^,^^5^ In the present study, we shed more light on the “recruitment” component of the task and showed that the deteriorative effects of aging were not limited to the “selection” component of ML-RT but also pertained to effector (limb) recruitment.

### Effect of age on CNS

#### Fiber density

Our results showed a significant reduction in fiber density within the SM1 voxel in association with aging. In general, this finding is in accordance with previous studies suggesting age-related decreases in white matter microstructural organization using tensor-based metrics.^13^^,^^14^^,^^15^^,^^16^ However, the tensor model fails to correctly model complex fiber geometries that happen to be present in up to 90% of white matter voxels,^51^ leading to difficulties with interpretation and limited biological specificity of the associated metrics.^52^ In contrast, the CSD-based model^19^ and its associated measures, such as fiber density, have been suggested to better deal with such limitations.^20^ Using CSD, previous studies also showed age-related decreases in fiber density.^4^^,^^25^ Although the more recent investigations on age-related differences in white matter using fiber density did not contradict findings obtained using tensor-based measures, fiber density measures have revealed greater specificity to such variations in major tracts of the brain.^53^ As fiber density relates to intra-axonal volume,^20^ the possible mechanisms underlying its reduction suggest a decrease in the number of fibers or shrinkage in the fiber diameter (or a combination thereof). Postmortem studies support this view as early studies reported a neuronal loss in aging.^54^ However, later investigations revealed conservation of the number of neurons in the older adults, yet provided evidence for cell shrinkage and synaptic density loss.^55^^,^^56^ More interestingly, the reduction in fiber density was observed only in the sensorimotor and not the occipital cortex which may indicate that the age-related decline in white matter microstructure is not uniform across the human brain.^57^

#### Concentrations of neurometabolites

Regarding NAA, although some studies have reported stable concentrations of this neurometabolite during the lifespan,^40^^,^^41^^,^^47^^,^^58^ our findings are consistent with more recent reports of decreases in NAA concentrations because of aging.^26^^,^^29^^,^^42^^,^^59^^,^^60^ NAA concentration has the largest peak in the MRS spectrum indicating its abundance in the brain and it appears to be involved in a wide variety of neural processes. Generally, NAA has been described as a marker of neural integrity.^29^^,^^33^^,^^35^ However, it has also been reported to be involved in other neural processes including (but not limited to) removing water from the cell as an osmolyte,^61^ oligodendrocyte/myelin synthesizing,^62^^,^^63^ partially sourcing energy for mitochondria,^64^ generation of acetylaspartylglutamate,^42^ and ligand gating for certain metabotropic glutamate receptors.^65^ Reduced NAA concentrations suggestively imply a dysfunction in the above-mentioned processes which can be potentially reflected in the secondary mechanisms, e.g., white matter microstructural changes. Similar to fiber density, the age-related reduction in NAA concentrations was found significant in the sensorimotor, and not the occipital cortex, suggesting spatial inhomogeneity of aging effects.^66^

We found no significant association between age and Cr concentrations in both sensorimotor and occipital cortex. This is in accord with previous work reporting stable concentrations of Cr across the human lifespan.^29^ Cr is thought to be involved in energy metabolism and its observed stability with age may reflect intact energy homeostasis in older adults. Accordingly, Cr has been used as an internal reference for the normalization of other neurochemicals.^67^ However, some studies have also reported an age-related increase^60^^,^^68^ or a decrease^7^^,^^69^ in Cr concentrations in various brain regions. These contradictory results might arise from several factors, such as differences in the studied region, methods of quantification, magnetic field strength, or sample size.

Regarding Cho, observation of increased concentrations of this neurometabolite as a function of age is a frequently reported finding in the literature.^29^^,^^60^^,^^68^ Cho is referred to as the membrane metabolite.^26^ Increased Cho-containing compounds may reflect the breakdown of phospholipid membranes,^70^ and it is normally linked to cell death. In this study, we found no significant association between age and Cho in both voxels. This observation could be tentatively interpreted to reveal that stable concentrations of Cho might reflect “cell death” as the less probable underlying neural mechanism of aging (at least in our sample). Relatedly, we might consider the observed reduction in fiber density as being mostly related to axonal diameter shrinkage rather than a global decrease in the number of axons. This is in accord with the postmortem histological studies reporting a preserved number of neurons in healthy aging.^55^

### Brain mediators of age-related differences in RT

Investigating the relation between behavioral and neural measures revealed that slower RT was significantly associated with reduced white matter fiber density, as well as lower concentrations of NAA, Cr, and Cho in the sensorimotor cortex. Similar to other findings of this study, the same associations were not observed in the occipital cortex, which served as the comparison voxel. Although we found associations between reaction speed and Cr as well as Cho concentrations in the sensorimotor cortex, they will not be further discussed here because these metabolites were not affected by aging and did not mediate the age-related differences in reaction speed.

Of interest, both fiber density and NAA concentrations in the sensorimotor voxel mediated the effect of age on reaction speed. Together these results suggest that aging negatively affects brain microstructural and neurochemical properties (NAA concentrations and fiber density) and this might compromise reaction speed in older adults. In addition, the significant path b in the indirect effect of the mediations pertains to the association between fiber density and/or NAA in the sensorimotor voxel and RT performance, controlling for the effect of age. This indicates that fiber density and/or NAA in the SM1 region can be generic predictors for RT even though aging may partially mediate this effect.

Decreased fiber density in association with aging has already been reported in major white matter tracts.^4^^,^^25^ This decrease may reduce the information transmission capacity of the brain which may be reflected in participants' reaction speed.^5^ In addition, previous studies in our lab reported associations between the properties of callosal motor fibers and response time in this multi-limb reaction time task.^71^ Using apparent axonal radius, axonal density, and fractional anisotropy we showed that the fibers connecting bilateral SM1 regions were positively associated with reaction performance. This further justifies the selection of SM1 for our investigation and underscores the importance of the structural integrity of the sensorimotor cortex for reaction time performance. Although the current study focused on WM microstructural and neurochemical properties in explaining age-related changes in MLRT performance, we acknowledge the potential contribution of other variables. For example, GM atrophy in nucleus accumbens and caudate has been shown to partially compromise action selection performance.^3^

Regarding NAA, several studies have found associations between concentrations of this neurometabolite and motor and/or cognitive performance.^7^^,^^72^^,^^73^ Here, we expanded evidence for these associations using a multi-limb reaction time task. Besides, NAA has been mostly referred to as a marker of neural integrity and it has been linked to WM-microstructural properties of the brain in normal aging and various pathologies.^33^^,^^34^^,^^74^^,^^75^ Hence, decreased NAA concentrations may be involved in structural neurodegeneration (as discussed next) and this might give rise to deficits in reaction speed in older adults.

### Three-way mediation (age, NAA, fiber density)

The above-mentioned findings led us to perform a 3-way mediation analysis in which NAA concentrations were considered as the mediator for the association between age and fiber density. Results of this analysis suggested that the age-related decline in concentrations of NAA in the sensorimotor voxel may have resulted in decreased fiber density in this voxel. However, these findings should be interpreted with caution and further research is required to validate this preliminary hypothesis.

NAA has been shown to be involved in myelination via releasing its aspartate group as well as acting as an osmolyte, preserving the cell volume and controlling the fluids across the membrane.^37^^,^^38^^,^^61^ Hence, the results of this three-way mediation analysis led us to conclude that the age-related reduction in NAA concentrations might result in the observed fiber density decline. Remarkably, our finding of a significant association in the indirect path (i.e., path b) of the mediation model indicated that NAA can also serve as a more generic predictor for FD in the SM1 voxel.

### Conclusion

Our findings indicate that both fiber density and NAA concentrations of the sensorimotor (but not occipital) cortex may act as potential mediators of the age-related decline in reaction speed. Thus, with advancing age, decreases in both fiber density and NAA concentrations may be linked with the slowing of reaction time which serves as a general proxy for information processing speed. Furthermore, the observed age-related reduction in fiber density is possibly mediated by a decline in NAA concentrations. Considering the role of NAA in various white matter-related processes, it is possible that the age-related decrease in the concentrations of NAA in the sensorimotor voxel compromises the microstructural properties of brain white matter, as reflected by a decline in fiber density and this, in turn, leads to a decline in reaction time in older adults. Longitudinal studies are required to confirm or reject this hypothesis.

### Limitations of the study

Although single voxel spectroscopy provides valuable information about the neurometabolic content of specific brain areas, it carries limitations in that other potentially relevant brain regions are not considered. With recent advancements in MRS data acquisition using multi-voxel spectroscopy, future studies can overcome these technological limitations. Nevertheless, the choice we made to look at the SM1 voxel in the left hemisphere is justified because its role in movement control in general and left, right, and bilateral limb movements specifically, is more critical than that of the right hemisphere SM1.^76^

Furthermore, we used a cross-sectional design to evaluate the associations among age, FD, and NAA. Accordingly, causal interactions cannot be inferred from the observed associations. Longitudinal study designs are more suitable to investigate the temporal sequence of the age-related changes in these measures.

Finally, although our dMRI pipeline included state-of-the-art processing techniques (such as 3-tissue CSD and the computation of FD) as well as pre-processing steps, continuous developments of these techniques will enable further improvements. One such step might be Rician bias correction (in addition to denoising),^77^ which is not yet part of the commonly implemented multi-tissue MRtrix3 pipeline at this time.

## STAR★Methods

### Key resources table

**Table:** 

REAGENT or RESOURCE	SOURCE	IDENTIFIER
**Software and algorithms**		

jMRUI v6.0	The MRUI Consortium	http://www.jmrui.eu/license-and-download/
SPID	Poullet et al.^78^	http://h/https://homes.esat.kuleuven.be/∼biomed/biosource/ManualSPID/node25.html
MRtrix3	Mrtrix	https://www.mrtrix.org/download/
FSL	FMRIB, Oxford	https://fsl.fmrib.ox.ac.uk/fsldownloads_registration
R (version 4.1.2, R Core Team, 2021)	R Foundation for Statistical Computing	https://cran.r-project.org/bin/windows/base/
ggplot2 package	Wickham^79^	https://cran.r-project.org/web/packages/ggplot2/index.html
SPM1	Karl Friston	http://www.fil.ion.ucl.ac.uk/spm/
MATLAB	The MathWorks, Natick, Massachusetts, USA	https://nl.mathworks.com/store/?gclid=CjwKCAjw0N6hBhAUEiwAXab-TeC0FFssJfawKmsFtp3jPv3t-4hkqq2RUrrSpmB2lA600ISHDDWzXRoCqFsQAvD_BwE&ef_id=CjwKCAjw0N6hBhAUEiwAXab-TeC0FFssJfawKmsFtp3jPv3t-4hkqq2RUrrSpmB2lA600ISHDDWzXRoCqFsQAvD_BwE:G:s&s_kwcid=AL!8664!3!462958882966!e!!g!!matlab%20download&s_eid=ppc_10961695282&q=matlab%20download

### Resource availability

#### Lead contact

Extra information and requests for codes and data should be directed to and will be fulfilled by the lead contact, Amirhossein Rasooli (amirhossein.rasooli@kuleuven.be).

#### Materials availability

This study did not generate new unique reagents.

### Experimental model and subject details

Healthy, right-handed participants were recruited (N=89). Thirteen participants were excluded due to low signal quality of MRS/dMRI data or inconsistent MRS voxel placement. The remaining participants (N=76) included 40 males, aged 20-75 years (mean ± std: 49.79 ± 16.86). They had no history of psychiatric or neuromuscular disorders and did not report intake of psychoactive medication. Moreover, all participants had normal or corrected-to-normal vision and passed the cutoff score of 24 on the Montreal Cognitive Assessment (MoCA) test.^80^ Besides, all the T1-weighted images were visually inspected by a radiologist for the presence of any abnormalities and, in case of any suspicious abnormalities, the participant was excluded. The study was approved by the local Medical Ethics Committee of KU Leuven (study number S58333) in accordance with the Declaration of Helsinki and its amendments. All participants provided written informed consent before the start of the study and received financial compensation for participating.

### Method details

#### Multi-limb reaction time (ML-RT) task

The participants performed an ML-RT task, as described in previous publications.^3^^,^^5^^,^^49^ They were seated in front of a screen, with their forearms resting on a table and their hands and forefeet on tablets with capacitive proximity switches (Figure 1A; Pepperl Fuchs CBN5-F46-E2, sampling frequency: 1000 Hz). Four squares representing the four limb segments were presented on the PC screen in logical order. Mapping of the visual stimuli was maximally congruent with the location of the effectors, i.e., the left and right upper squares represented the left and right hands, whereas the left and right lower squares represented the left and right feet, respectively. Once all four limb segments were in contact with the tablets simultaneously, a subset of the squares on the screen turned blue, indicating the limbs that should be moved (Figure 1B). In response to this stimulus, participants had to release contact with the corresponding tablet(s) as quickly and as correctly as possible by lifting the indicated limb segment(s). All 15 possible coordination modes were tested and arranged in 6 coordination clusters (Figure 1C) composed of 4 single-limb modes (1L); 2 homologous upper or lower limb stimulus modes (2L-Homo); 2 ipsilateral (right upper right lower or left upper left lower) limb stimulus modes (2L-Ipsi); 2 diagonal (left upper right lower or left lower right upper) limb stimulus modes (2L-Diag); four 3-limb (2L-Ipsi plus upper/lower contralateral) stimulus modes (3L); and one 4-limb mode (4L).

The experimental procedure has been described in detail in previous work.^49^ After familiarization, participants performed randomized 10-trial runs of each of the 15 stimulus modes (150 trials in total). At the beginning of each run, the image of the stimulus that would appear on the screen for the next 10 trials was shown (predictive: no choice required). The time interval between the stimulus onset and lifting up the corresponding limb(s) in error-free trials was defined as reaction time (RT). For each participant, trials with RT values >3 scaled median absolute deviations^81^ within each stimulus mode of each participant were considered outliers and removed (1.1 ± 1.3 (mean ± SD) of trials across all participants). The median value of the remaining trials in each stimulus mode was obtained and averaged to summarize the initial 15 stimulus modes into 6 stimulus clusters (1L, 2L-Homo, 2LIpsi, 2L-Diag, 3L, and 4L). As was shown in previous work by our group,^3^^,^^5^^,^^49^ the 2L-Diag and 3L stimulus clusters are more difficult to perform than the other stimulus clusters which lead to higher inter-subject variability in RT for these clusters. Thus, we used the average RT in 2L-Diag and 3L stimulus clusters as our final measure of motor performance.

#### ^1^H-magnetic resonance spectroscopy (^1^H-MRS)

^1^H-MRS data were acquired using a Philips 3T Achieva magnetic resonance scanner (Philips Healthcare, The Netherlands) with a 32-channel receiver head coil, located at the University Hospital of KU Leuven, Belgium. A high-resolution three-dimensional T1-weighted structural image was acquired (three-dimensional transient field echo (TFE); repetition time (TR) = 9.6 ms; echo time (TE) = 4.6 ms; inversion time (TI) = 900 ms; flip angle = 8°; voxel size = 0.98 × 0.98 × 1.2 mm^3^; field of view = 250 × 250 × 192 mm^3^; 160 coronal slices). ^1^H-MRS spectra were acquired from two voxel locations in the visuomotor system, namely the left sensorimotor cortex (SM1) (Figure 2A) and occipital cortex (Occ) (Figure 2B). MRS data were acquired using a point resolved spectroscopy (PRESS) sequence (TR = 2000 ms, TE = 22 ms, number of averages (NA) = 128, scan duration ∼4.27 min, spectral bandwidth = 2000 Hz, data size = 1024 points) with excitation water suppression. The voxel size was 1.5 × 1.5 × 1.5 cm^3^ in both the SM1 and Occ cortex voxels. The SM1 voxel was centered over the left hand-knob, parallel to the anterior and posterior axis,^82^ and was rotated in the coronal and sagittal planes to align with the external surface of the brain.^83^ The Occ voxel was centered on the median line, aligned with the cerebellar tentorium in the sagittal plane, and positioned as posteriorly as possible. For absolute metabolite quantification, the unsuppressed water signal was also acquired using the same acquisition parameters, except for the number of averages (NA= 16).

MR spectra were processed using jMRUI v6.0^84^ and the in-house developed software SPID.^78^ All spectra were phase- and frequency-corrected prior to quantification. Hence, spectral registration was performed to align all the spectra in order to correct for participants' motion artifacts retrospectively. jMRUI QUEST was utilized to determine the signal-to-noise ratios (SNR) in the time domain (maximum of FID/standard deviation of FID tail). Spectra with linewidths more than 10 Hz or SNR less than 5 were excluded from quantification and further processing. All spectra were visually inspected to ensure the absence of artifacts (i.e., spurious echoes, and lipid contamination). Metabolite signals and corresponding non-suppressed water signals were quantified using peak integration in SPID. Water-referenced concentrations of NAA, Cr, and Cho were quantified for each voxel location. Cramer-Rao lower bound (CRLB) values were calculated and only the metabolites with CRLB values below 20% were included in the statistical analysis. The T1-weighted MR images, acquired for the localization and placement of the MRS voxels were segmented with a statistical parametric mapping approach using SPM12 (http://www.fil.ion.ucl.ac.uk/spm/). Voxel registration was performed using custom-made scripts developed in MATLAB (The MathWorks, Natick, Massachusetts, USA) by Dr. Nia Goulden (Bangor University Wales, UK),.^85^ Using the T1-weighted MR image and the orientation and location information from the Philips SPAR files, the scripts generated a binary mask of the voxel location, which was then used to calculate the partial volumes of gray matter (GM), white matter (WM), and cerebrospinal fluid (CSF) within the voxel. The segmented tissue volumes were then used to correct for metabolite concentrations and quantified using SPID for differences in CSF content according to.^86^ T1 and T2 values used in our study were 1331 ms, 832 ms, and 3817 ms (T1) and 110 ms, 79 ms, and 503 ms (T2) for GM, WM, and CSF, respectively. Metabolite relaxation times that were used for calculating the final corrected metabolite concentrations were taken from previous studies (T1 relaxation times: NAA = 1.34 ms, Cr = 1.11 ms, Cho = 1.14 ms; T2 relaxation times in SM1: NAA = 247 ms, Cr = 162 ms, Cho = 222 ms; T2 relaxation times in Occ: NAA = 301 ms, Cr = 178 ms, Cho = 222 ms).^87^^,^^88^

#### Diffusion magnetic resonance imaging (dMRI)

Multishell dMRI data were acquired with the same scanner used for MRS. A spin-echo EPI sequence was used, with the following parameters: b = 700 s/mm^2^ (16 directions); b = 1200 s/mm^2^ (30 directions); b = 2800 s/mm^2^ (50 directions); six interleaved volumes without diffusion weighting (b = 0 s/mm^2^); voxel size = 2.5 × 2.5 × 2.5 mm^3^, TE/TR = 74/9000 ms; SENSE = 2; matrix size = 96 × 96 (all of the images were reconstructed without zero padding); and number of axial slices = 50. In addition, one b = 0 s/mm^2^ image was acquired with reversed phase encoding, for the purpose of susceptibility-induced (EPI) distortion correction.

All the dMRI processing steps in this study were conducted either using commands implemented within MRtrix3^89^ or using MRtrix3 scripts that interfaced with external software packages such as FSL,^90^ as described below. In brief, dMRI data were denoised^91^ and corrected for eddy current, motion, and susceptibility-induced distortions.^92^^,^^93^^,^^94^ Afterward, 3-tissue response functions representing single-fiber white matter, grey matter, and CSF were obtained from the data themselves using an unsupervised approach.^95^ Subsequently, dMRI data were upsampled to an isotropic voxel size of 1.3 mm (to improve downstream fiber orientation distribution registration^20^^,^^96^ and a brain mask was estimated. Next, 3-tissue constrained spherical deconvolution (CSD) was performed for each subject, using the averaged (across all subjects) response functions for each tissue type with the multi-shell multi-tissue CSD algorithm^51^ resulting in the white matter fiber orientation distribution (FOD) for each voxel. Joint bias field correction and global intensity normalization of the 3-tissue parameters were performed in the log-domain.^97^

To achieve spatial correspondence, an iterative registration and averaging approach was used to generate a study-specific FOD template to which all 76 participants’ FODs were registered.^98^^,^^99^ Then, an apparent fiber density (FD) map was obtained for each participant across all brain voxels in the study-specific template space.

For each participant, the T1-weighted image was rigidly registered to the b=0 volume of corrected dMRI data and the corresponding transform was combined with the warp obtained previously from the registration of participant’s FOD to the study-specific template space. Using the combined transform, the WM mask obtained from the pre-processing of MRS data was transformed to the FOD template space with linear interpolation. Then a threshold of 0.5 was applied to the resulting image (in order to result again in a binary mask, after linear interpolation of the original binary voxel mask) and the mean FD was calculated in the obtained WM binary mask.

### Quantification and statistical analysis

#### Bivariate correlation analysis

The associations between age and measures of reaction time, neurochemical concentrations, and WM microstructure, were investigated using bivariate correlation analysis. Due to the non-normal distribution of data, non-parametric Spearman’s correlation coefficients (ρ) were obtained. Since gender was found to have a negligible effect on the evaluated associations, it was not considered for further investigations. Statistical analyses were conducted in R (version 4.1.2, R Core Team, 2021) and figures were produced using the package ggplot2.^79^ P values were corrected for multiple comparisons using Bonferroni’s method.^100^

#### Mediation analysis

Three mediation relations were tested for their significance.^101^ In the first two models, the 3-way association between age, brain neurochemical concentrations or microstructural metrics, and reaction speed (i.e., RT) were investigated (Figure 3A). We hypothesized that aging negatively affects brain measures (i.e., reduced FD and WM-related neurometabolites) and this may mediate higher RT with aging. In the last model, the 3-way association between age, neurochemical concentrations, and WM microstructure were investigated (Figure 3B). Specifically, we hypothesized that aging leads to a reduction in concentrations of the neurometabolites that are related to WM and this may mediate FD decline in aging. The commonly used mediation model,^102^ implemented in the M3 mediation toolbox,^103^^,^^104^ was used (see Figure 3 for details). Bootstrapping (n=10,000) was used to determine the bias-corrected confidence interval (CI) for the mediation effect (ab). Accordingly, mediation effects with 95% CIs entirely above zero (one-tailed test based on the directional hypotheses) were regarded as significantly mediating the relation between independent and dependent variables in all models.

## Data Availability

• All the reported data in this study can be shared by the lead contact upon request. • The study does not generate any original code. • Upon request, the lead contact can provide any additional information necessary to reanalyze the reported data.
