# Supplementary material for: White matter and neurochemical mechanisms underlying age-related differences in motor processing speed
Source: iScience. 2023 May 3;26(6):106794. doi: 10.1016/j.isci.2023.106794 (PMC10225899; doi:10.1016/j.isci.2023.106794)
Supplement: Document S1. Figure S1 [file mmc1.pdf]

iScience, Volume *26*

Supplemental information

White matter and neurochemical mechanisms underlying age-related differences in motor processing speed

Amirhossein Rasooli, Hamed Zivari Adab, Peter Van Ruitenbeek, Akila Weerasekera, Sima Chalavi, Koen Cuypers, Oron Levin, Thijs Dhollander, Ronald Peeters, Stefan Sunaert, Dante Mantini, and Stephan P. Swinnen

Supplemental materials

Figure S1

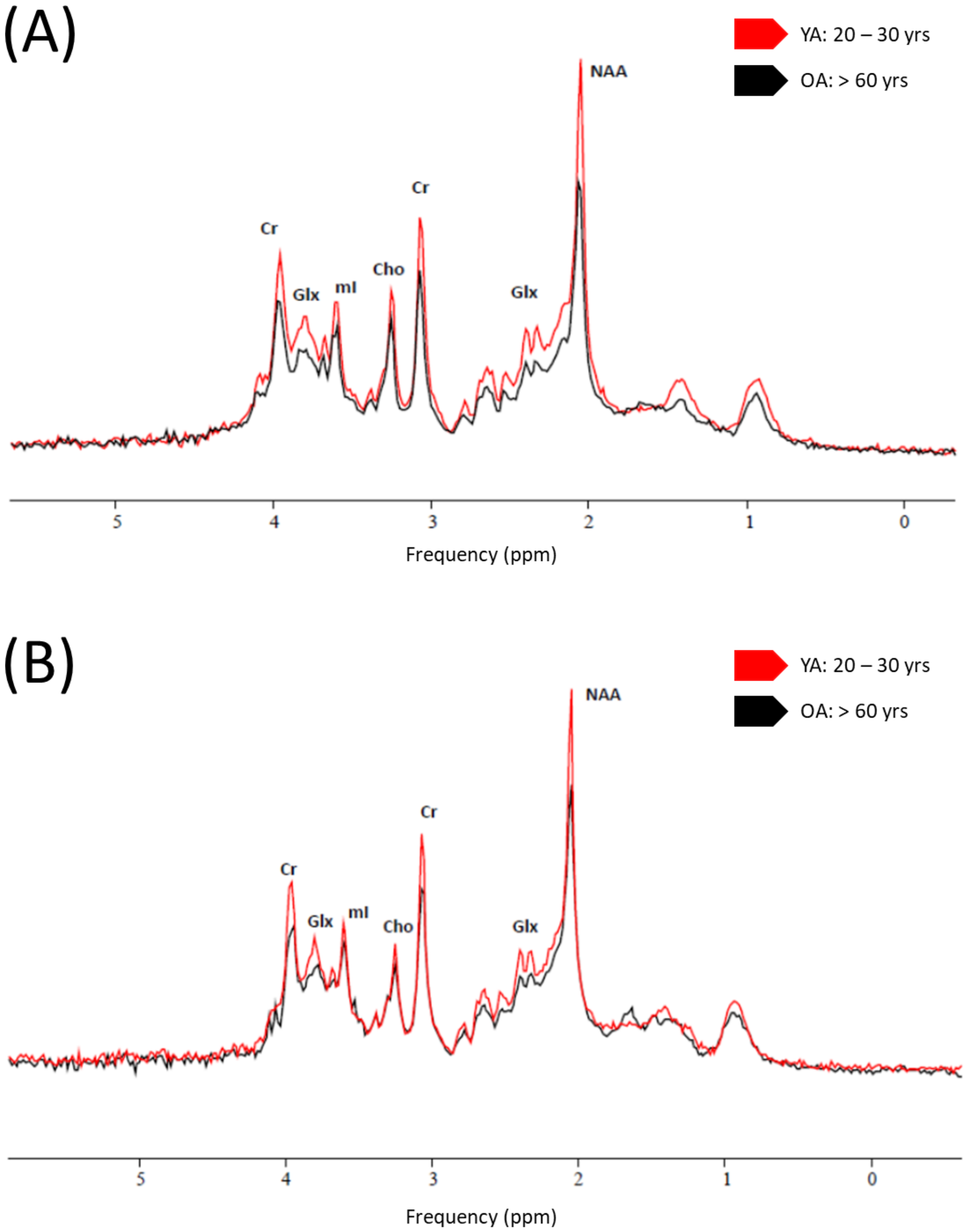

Figure S1. Average spectrum in the two extreme groups (YA: 20 – 30 yrs and OA: > 60 yrs), it is related to the Figure 2 in the STAR Methods. (A) results in sensorimotor, and (B) results in occipital voxels.
